# Dr. C. Ernest Campbell

**Published:** 1915-01

**Authors:** 


### OBITUARY

Readers are requested to report promptly the death of all physicians in Western New York, or former residents of this region, or graduates of any medical school in Western New York, and to notify the families of the deceased of our desire to publish adequate obituary notices.

**Dr. C. Ernest Campbell,** N. Y. Homoeopathic College 1884, born in Painted Post 55 years ago, was found dead in his office November 23, 1914. Death had evidently occurred several days previously, presumably from apoplexy.

